# A Coverage Optimization Approach for Wireless Sensor Networks Using Swarm Intelligence Optimization

**DOI:** 10.3390/biomimetics10110750

**Published:** 2025-11-06

**Authors:** Shuxin Wang, Qingchen Zhang, Yejun Zheng, Yinggao Yue, Li Cao, Mengji Xiong

**Affiliations:** 1 School of Intelligent Manufacturing, Shanghai Zhongqiao Vocational and Technical University, Shanghai 201514, China; 2 School of Intelligent Manufacturing and Electronic Engineering, Wenzhou University of Technology, Wenzhou 325035, China; 3 Engineering Technology Department, Shanghai Caoyang Vocational School, Wenzhou 200065, China

**Keywords:** wireless sensor networks, flamingo search optimization algorithm, chaotic sequence, coverage rate, coverage optimization

## Abstract

WSN coverage optimization faces two key challenges: firstly, traditional algorithms are prone to getting stuck in local optima, leading to ‘coverage holes’ in node deployment; Secondly, in dynamic scenarios (such as imbalanced energy consumption of nodes), the convergence speed of the algorithm is slow, making it difficult to maintain high coverage in real time. This study focuses on the coverage optimization problem of wireless sensor networks (WSNs) and proposes improvements to the Flamingo Search Optimization Algorithm (FSA). Specifically, the algorithm is enhanced by integrating the elite opposition-based learning strategy and the stagewise step-size control strategy, which significantly improves its overall performance. Additionally, the introduction of a cosine variation factor combined with the stagewise step-size control strategy enables the algorithm to effectively break free from local optima constraints in the later stages of iteration. The improved Flamingo Algorithm is applied to optimize the deployment strategy of sensing nodes, thereby enhancing the coverage rate of the sensor network. First, an appropriate number of sensing nodes is selected according to the target area, and the population is initialized using a chaotic sequence. Subsequently, the improved Flamingo Algorithm is adopted to optimize and solve the coverage model, with the coverage rate as the fitness function and the coordinates of all randomly distributed sensing nodes as the initial foraging positions. Next, a search for candidate foraging sources is performed to obtain the coordinates of sensing nodes with higher fitness; the coordinate components of these candidate foraging sources are further optimized through chaos theory to derive the foraging source with the highest fitness. Finally, the coordinates of the optimal foraging source are output, which correspond to the coordinate values of all sensing nodes in the target area. Experimental results show that after 100 and 200 iterations, the coverage rate of the improved Flamingo Search Optimization Algorithm is 7.48% and 5.68% higher than that of the original FSA, respectively. Furthermore, the findings indicate that, by properly configuring the Flamingo population size and the number of iterations, the improved algorithm achieves a higher coverage rate compared to other benchmark algorithms.

## 1. Introduction

Wireless Sensor Networks (WSNs), as an advanced information collection and processing technology, have emerged as a top-priority research frontier in the 21st century [1]. Composed of numerous small-sized, cost-effective, and low-power sensor nodes, WSNs form a multi-hop network system via wireless communication [2,3]. This unique network structure supports complex large-scale monitoring and tracking services across diverse fields, such as target tracing, intrusion detection, weather monitoring, disaster response, and battlefield reconnaissance [4,5]. According to the International Telecommunication Union (ITU), the global Internet of Things (IoT) ecosystem encompassed 14.6 billion devices in 2023, with 40% of these IoT deployments relying on WSN infrastructure. However, key challenges persist in conventional coverage optimization algorithms: In smart agriculture, existing methods struggle to adapt to dynamic and complex environments. Traditional algorithms typically demand accurate geographic location information of sensor nodes, which not only increases hardware costs but also consumes excessive energy—leading to premature network energy depletion, incomplete farmland coverage, and inaccurate data collection. Similarly, in disaster monitoring (where rapid deployment and real-time data transmission are critical), traditional methods exhibit more pronounced limitations: Reliance on preconfigured location information may delay network establishment, and high coverage vulnerability rates can result in the loss of critical disaster signals, thereby undermining early warning system effectiveness.

Current research on WSN coverage optimization primarily focuses on reducing energy consumption and enhancing monitoring quality through techniques like node scheduling and dynamic topology control [6]. While these solutions show potential, most still require sensor node geographic location information, introducing additional challenges related to hardware costs and energy usage [7]. This gap highlights the significant research value of developing a coverage optimization algorithm independent of geographic location data. The theoretical foundation of WSN coverage optimization includes three core aspects: coverage models, optimization objectives, and constraints. Coverage models—the basis of optimization—describe how sensor nodes detect and cover target areas [8]. Scholars have proposed various models, such as probabilistic and deterministic coverage models. Probabilistic models [9] account for inherent uncertainties in real-world WSN deployments, treating sensor detection capabilities as probabilistic events. By statistically analyzing the probability of a node detecting a target within its range, these models are well-suited for dynamic, unpredictable environments (e.g., wildlife tracing, where sensor readings are prone to environmental interference). In contrast, deterministic models [10] employ geometric and analytical approaches, assuming the precise knowledge of sensor parameters. They define clear boundaries for sensor sensing ranges and use mathematical algorithms to ensure comprehensive, accurate coverage—making them ideal for structured scenarios like factory floor monitoring, where predictable sensor behavior is required. Though conceptually distinct, these models complement each other, providing a holistic theoretical framework for subsequent algorithmic and experimental research, as well as tools for designing application-specific coverage strategies.

Optimization objectives, the core of WSN coverage optimization, dictate algorithm design directions and performance [11]. Common objectives—maximizing coverage area, minimizing node count, and optimizing node distribution—serve distinct purposes in different contexts. Maximizing coverage ensures full target area monitoring, critical for applications like environmental disaster early warning (where blind spots cause severe consequences). Minimizing nodes reduces hardware costs and energy consumption, a priority for large-scale deployments emphasizing economic and energy efficiency. Optimizing node distribution aims for uniform node arrangement, improving data collection uniformity and balancing network load—vital for precision agriculture. However, these objectives face context-dependent trade-offs: For example, in security surveillance WSNs, maximizing coverage may take precedence over minimizing nodes. Further, objectives must be pursued under constraints like limited energy, restricted communication ranges, and constrained computational capabilities [12,13]. This necessitates careful trade-off analysis during algorithm design to balance conflicting goals—a key motivation for this study, as addressing these unresolved challenges is essential to advancing WSN practical applicability.

The research on WSN coverage optimization leveraging the Flamingo Search Optimization Algorithm endeavors to tackle the pressing challenges of energy consumption, monitoring quality, and network lifespan. By emulating the natural behaviors of flamingos, such as their coordinated foraging patterns and group migration strategies, this study innovatively translates biological intelligence into computational solutions [14]. In nature, flamingos efficiently allocate resources and adapt to environmental changes through collective decision-making. Analogously, the proposed algorithm enables sensor nodes to dynamically adjust their operational states and spatial configurations. It achieves the dual goals of minimizing energy consumption, by reducing unnecessary node activities, and maximizing monitoring quality, through intelligent node collaboration. This approach represents a significant departure from traditional methods, which often rely on static or heuristic-based strategies. As a result, it offers a novel solution for WSN applications, breaking through the limitations of existing algorithms. The research not only enriches the theoretical body of knowledge by introducing a new bio-inspired optimization paradigm but also holds broad application prospects. It has the potential to revolutionize WSN deployments in various fields, from remote environmental monitoring to industrial IoT systems, by providing more energy-efficient, reliable, and high-performance network solutions.

The investigation into WSN coverage optimization based on the Flamingo Search Optimization Algorithm carries profound theoretical and practical implications [15,16]. By introducing novel intelligent optimization algorithms, this research aims to resolve the existing issues in WSN coverage optimization, enhance network performance, and enable more efficient monitoring and data transmission. It not only facilitates the expansion of the Flamingo Search Optimization Algorithm within the communication domain but also provides innovative ideas and methodologies for WSN optimization. Through the optimization of coverage strategies, the energy consumption of sensor nodes can be reduced, the network lifespan extended, and the monitoring quality improved concurrently, thus delivering more reliable and stable network services across various application scenarios. Moreover, this research involves the cross-integration of multiple disciplines, fostering communication and collaboration among different fields and driving the innovation and development of related technologies. Therefore, this study holds significant research value and promising practical application prospects.

The following are this paper’s primary contributions:

1. Propose an MIFSA hybrid algorithm with enhanced global exploration via an elite reverse learning strategy.
2. Design a multi-stage adaptive parameter strategy to dynamically balance search accuracy and speed.
3. Validate the algorithm’s performance in 23 benchmark functions and wireless sensor network coverage optimization, outperforming 7 comparison algorithms.

The organization of the subsequent sections in this paper is as follows: Section 2 introduces the mathematical model for the coverage of wireless sensor network. Section 3 provides a detailed introduction to the flamingo search optimization algorithm. Section 4 elaborates on the improved flamingo search optimization algorithm (MIFSA). Section 5 presents the simulation results of the MIFSA and seven other algorithms. Finally, the paper concludes with conclusions and future prospects.

## 2. Mathematical Model for the Coverage of Wireless Sensor Network

(1) Sensing Model

To reduce the complexity of the sensing model, this problem is often simplified to a binary model, that is, the Boolean sensing model [17]. In a two-dimensional plane, the sensing range of a sensor node is a circular area with the sensor node as the center and radius *R*. The probability of being sensed within this range is 1, and the area beyond this range is considered non-sensable with a probability of 0. The mathematical model is as follows [18,19]:

(1) $$P(r,s_{i})=\left\{\begin{array}{lll} 1, & if & d(r,s_{i})\leq R\\ 0, & & otherwise \end{array}\right.$$

where $P(r,s_{i})$ represents the probability that sensor $d(r,s_{i})$ represents the Euclidean distance from the sensor to the point. the variable $d(r,s_{i})$ in the vicinity of Equation (1) as the Euclidean distance between node rand sensor *s_i_*, to avoid any ambiguity.

(2) Coverage Rate

**Figure 1:**
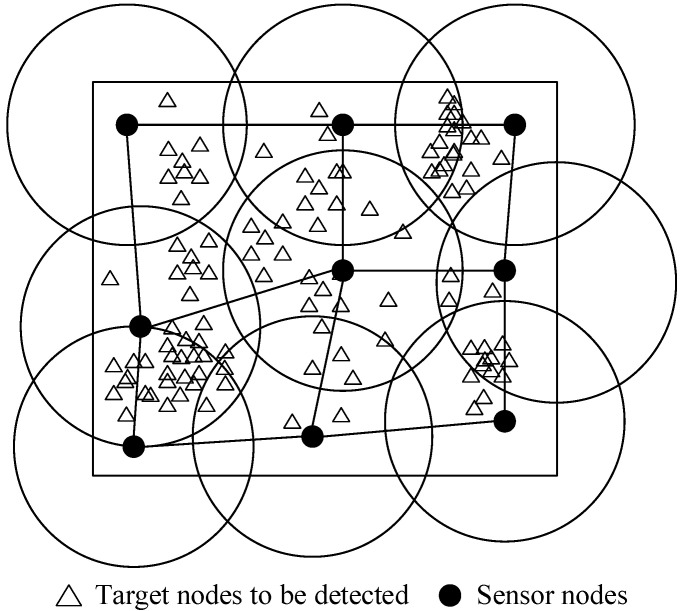
Schematic diagram of coverage effect of wireless sensor network.

Most of the research on coverage strategies of wireless sensor networks is based on regional grids to solve the regional coverage rate [20,21]. The model is shown in Figure 1. The regional coverage rate of the sensor network is a commonly used quantitative index and the basis for comparing the advantages and disadvantages of optimization algorithms [22].

Suppose there are *n* sensors in the sensor network, and its set is expressed as:

(2) $$S\;=\;\left\{s_{i}\right\},i=1,\cdots ,n,$$

where *S* is the sensor network, *s_i_* is a sensor node, and *n* is the number of sensor nodes in the network.

The regional coverage rate is defined as the ratio of the effective coverage area of all sensor nodes in the target area to the total area of the target area, as shown in the following formula [23,24].

(3) $$\eta =\frac{\sum_{i=1,\cdots ,n}\Phi_{s_{i}}}{A}$$

where η is the regional coverage rate of the sensor network, $\Phi_{s_{i}}$ is the sensing area of the *i*-th sensor, and *A* is the total area of the target area [25].

A point *r* in the target area may be sensed by different nodes in the network. Then the probability that point *r* is sensed by the sensor network is

(4) $$P(r,S)=1-\prod_{i=1,\cdots ,n}\left[1-P(r,s_{i})\right]$$

Suppose there are *m* points in the area, then the final total coverage rate of the sensor network for all sensed points in the area is [26]:

(5) $$C(s)=\frac{\sum_{i=1,\cdots ,m}P(r,S)}{A}$$

where C(s) is the total coverage rate of the sensor network *S* for all sensed points in the target area [27].

The optimization objective of this paper is to deploy a certain number of sensor nodes in the target area. By applying different swarm intelligence algorithms to optimize the node layout, the coverage rate of the wireless sensor network C(s) is improved to the best value [28].

## 3. Flamingo Search Optimization Algorithm

The Flamingo Search Optimization Algorithm (FSA) belongs to a category of meta-heuristic optimization methods inspired by the migration and foraging behaviors of flamingo colonies, as cited in relevant academic literature [29,30]. Taking inspiration from how flamingos convey information about food sources through vocal signals and adjust their spatial distributions during group activities [31], the FSA simulates the dynamic interactions among flamingos within an unknown search space. By iteratively updating the positions of individual flamingo-inspired agents, the algorithm explores the solution domain in a systematic manner to identify the global optimal solution. In terms of structure, FSA consists of two core phases: beak scanning (which represents the local exploitation of promising regions) and foot movement (which denotes the global exploration of new areas). This dual-phase framework enables the algorithm to maintain search diversity in the initial stages while guaranteeing rapid convergence in later iterations, making it highly suitable for addressing complex high-dimensional nonlinear optimization problems in fields such as engineering, data science, and computational modeling [32,33].

Flamingos are social migratory birds that primarily feed on algae, shrimp, and other similar organisms, and they exhibit a unique feeding method: they bend their long necks downward, invert their heads, and then move slowly while sweeping their curved beaks through the water and touching the seabed or riverbed to capture food [34]. Flamingos display two key types of behavior: foraging behavior and migratory behavior. The entire flamingo population typically resides in areas with ample food resources. When the food density in their inhabited area can no longer satisfy the population’s feeding needs after prolonged foraging, the flamingos will initiate migration [35]. Flamingos in different locations notify their peers of their current positions and the availability of food in their respective areas through mutual vocalizations. The flamingo population lacks prior knowledge of the location with the highest food concentration in the current search area; instead, they discover positions with more abundant food than previously known areas by continuously updating the positions of individual group members [36].

The position update of a single flamingo particle in the Flamingo Search Algorithm is mainly based on the foraging and migratory behaviors of flamingos. When searching for the global optimal solution in a certain search space, since the position of the global optimal solution is unknown, flamingo particles search the space and develop through information exchange and fixed position—moving behaviors among each other, and finally obtain the optimal solution [37]. The foraging behavior of flamingos includes three behavioral characteristics: communication behavior, beak-scanning behavior, and biped-moving behavior. The communication behavior helps a single flamingo particle know the position of the flamingo with the most food in the flamingo population [38]. Then, while scanning for food with the beak, the biped of the flamingo moves towards the position of the flamingo with the richest food in the population. In the t-th iteration, the moving distance of a foraging flamingo is the sum of the biped-moving distance and the beak-scanning range. The specific formula is as follows:

(6) $$b_{ij}^{t}=\varepsilon_{1}\times xb_{j}^{t}+G_{2}\times \left|G_{1}\times xb_{j}^{t}+\varepsilon_{2}\times x_{ij}^{t}\right|$$

where *xb_j_^t^* is the position of the flamingo particle with the best fitness value in the *j*-th dimension during the *t*-th position iteration of the flamingo population. *G*1 and *G*2 are random numbers following the standard normal distribution. *ε*1 and *ε*2 take values of −1 or 1, which are randomly determined during the algorithm operation to increase the search range of flamingo foraging and quantify individual differences [39]. *x_ij_^t^* is the position of the *i*-th flamingo in the *j*-th dimension in the *t*-th iteration.

The specific formula for updating the position of a foraging flamingo is as follows:

(7) $$x_{ij}^{t+1}=(x_{ij}^{t}+b_{ij}^{t})/K$$

where *x_ij_^t+^*^1^ is the position of the *i*-th flamingo in the *j*-th dimension in the (*t* + 1)-th iteration. *K* is a random number following the chi-square distribution with *n* degrees of freedom and is used as a diffusion factor in the algorithm to expand the foraging range of flamingos [40].

In the Flamingo Search Algorithm, the foraging flamingo particles play a major role in local search and optimization in the position update of the entire flamingo population, strengthening the exploration of the local area. When the food in the current search area is scarce, that is, the fitness of the flamingo particles in the current area is low, some flamingos in the population will migrate to another area with more abundant food for exploration [41,42]. The migratory behavior of the flamingo population reflects its global optimization ability. In each iteration, a certain proportion of the flamingo population will migrate. The formula for updating the position of migratory flamingos is as follows:

(8) $$x_{ij}^{t+1}=x_{ij}^{t}+\omega \times (xb_{j}^{t}-x_{ij}^{t})$$

where *x_ij_^t+^*^1^ is the position of the *i*-th flamingo in the *j*-th dimension in the (*t* + 1)-th iteration. The parameter *t* is the position of the flamingo particle with the best fitness value in the *j*-th dimension during the *t*-th position iteration of the flamingo population. The parameter *ω* is a Gaussian random number with *n* degrees of freedom, which is used to expand the search space during the flamingo migration and simulate the randomness of individual behaviors during a specific migration [43].

The communication behavior allows flamingos to guide the group to update positions by transmitting information about food-rich positions through calls, even if individuals do not fully know the global optimal position. The beak-scanning behavior simulates the more intensive search of flamingos in food-rich areas, adjusting the scanning range and frequency through beak and foot movements. The combination of these two behaviors enables the FSA to effectively find the optimal solution in uncertain and dynamic environments, balancing global exploration and local exploitation [44].

The moving step of flamingo foraging is the sum of the beak-scanning range and the foot-moving distance:

(9) $$b_{ij}^{t}=\varepsilon_{1}\times xb_{j}^{t}+G_{2}\times \left|G_{1}\right.\times xb_{j}^{t}+\varepsilon_{2}\times \left.x_{ij}^{t}\right|$$

Update the position of flamingo foraging behavior.

(10) $$x_{ij}^{t+1}=(x_{ij}^{t}+\varepsilon_{1}\times xb_{j}^{t}+G_{2}\times \left|G_{1}\times xb_{j}^{t}+\varepsilon_{2}\times x_{ij}^{t}\right|)/K$$

When the food in the current foraging area is scarce, the flamingo population will migrate to the next area with more abundant food.

(11) $$x_{ij}^{t+1}=x_{ij}^{t}+\omega \times (xb_{j}^{t}-x_{ij}^{t})$$

where *xb_j_* represents the *j*-th dimensional position of the flamingo with the best fitness in the population in the *t*-th iteration, and *ω* = *N*(0, *N*) is a Gaussian random number with *N* degrees of freedom, which is used to increase the search space during the flamingo migration and simulate the randomness of individual behaviors during a specific migration.

## 4. Improved Flamingo Search Optimization Algorithm

(1) Elite opposition-based learning strategy

The elite opposition-based learning strategy is applied to the initialization stage. Through the generation of opposite solutions and the selection of elite individuals, not only can the search range of the algorithm be expanded, improving the global search ability, but also the ability of the algorithm to avoid local optima can be enhanced [45,46].

(12) $${\left(x_{ij}^{\prime }\right)}^{t}=k(lb_{j}^{t}+ub_{j}^{t})-x_{ij}^{t}$$

(2) Stagewise step-size control strategy

A spiral search strategy based on the warning value is proposed to expand the search range while approaching the optimal solution. To improve the search accuracy and convergence speed, a non-linear attenuation factor *μ* is introduced, enabling the algorithm to search different regions widely in the initial stage and focus on optimizing the known regions in the middle and later stages [47,48]. The updated moving distance of the improved flamingo is

(13) $$X_{i,j}^{t+1}=\left\{\begin{array}{l} xb+|x_{i,j}^{t}-xb_{j}^{t}|\times e^{t}\times \cos(2\pi l),R<0.5\\ x_{i,j}^{t}+(xb_{j}^{t}-x_{i}^{t})\times w\times \mu \end{array}\right.$$

The parameters are expressed as

(14) $$\begin{array}{l} l=(a-1)\times rand+1\\ a=\left(-\frac{t}{iter_{\max}}\right)-1\\ \mu =\frac{1}{a\times \frac{t}{iter_{\max}}}e \end{array}$$

(3) Chaotic cosine variation factor

After flamingos find food, a large number of them gather, which may cause the algorithm to fall into local optima and reduce the diversity of the algorithm. A chaotic cosine variation factor is introduced into the position update of flamingo foraging [49]. By adjusting in different stages, the extensive exploration of unknown regions by flamingos is strengthened, and the probability of falling into local optima is reduced.

(15) $$\eta =e^{\delta \times \cos\left(\frac{\pi }{2}u\right)},\;u=1-\frac{t}{iter_{\max}}$$

(4) Generation of relevant parameters using circle chaotic map [50].

(16) $$y_{i+1}=\mathrm{mod}\left(y_{i}+0.2-\left(\frac{0.5}{2\pi }\right)\sin(2\pi y_{i}),1\right)$$

The improved flamingo position update.

(17) $$x_{i,j}^{t+1}=\left\{\begin{array}{l} Q\times \exp\left(\frac{xw_{j}^{\prime }-x_{i,j}^{\prime }}{i^{2}}\right),i>\frac{n}{2}\\ xb_{j}^{t+1}+|x_{i,j}^{\prime }-xb_{j}^{t+1}|\times \\ L\times A^{+}\times \eta \times \cos(2\pi k),i\leq \frac{n}{2} \end{array}\right.$$

Combining Formulas (16) and (17) to improve the strategy, the updated foraging position of the flamingo is as follows

(18) $$Y_{ij}^{t+1}=(x_{ij}^{t}+\varepsilon_{1}x\times b_{j}^{t}+G_{2}\times |G_{1}x\times b_{j}^{t}+\varepsilon_{2}\times x_{ij}^{t}|)\times \eta x\cos(2\pi k)$$

(5) Adaptive selection mechanism of Levy flight

An adaptive selection mechanism of Levy flight strategy is proposed [51]. Through an adaptive factor p that decreases with the number of iterations, sparrow individuals are randomly selected for Levy flight perturbation to enhance the diversity of sparrow positions. The adaptive selection factor [52]:

(19) $$p=1-\frac{t}{iter_{\max}}e^{\frac{iter_{\max}-t}{iter_{\max}}}$$

The position update of Levy flight is

(20) $$\begin{array}{c} {\left(x_{i}^{\prime }\right)}^{t}=x_{i}^{t}+m⊕Levy(\lambda ) \end{array}$$

The pseudocode of Multi-strategy Improved Flamingo Search Algorithm (MIFSA) algorithm is shown in Algorithm 1. Initialize parameters such as *P* and calculate the fitness values.

**Figure 2:**
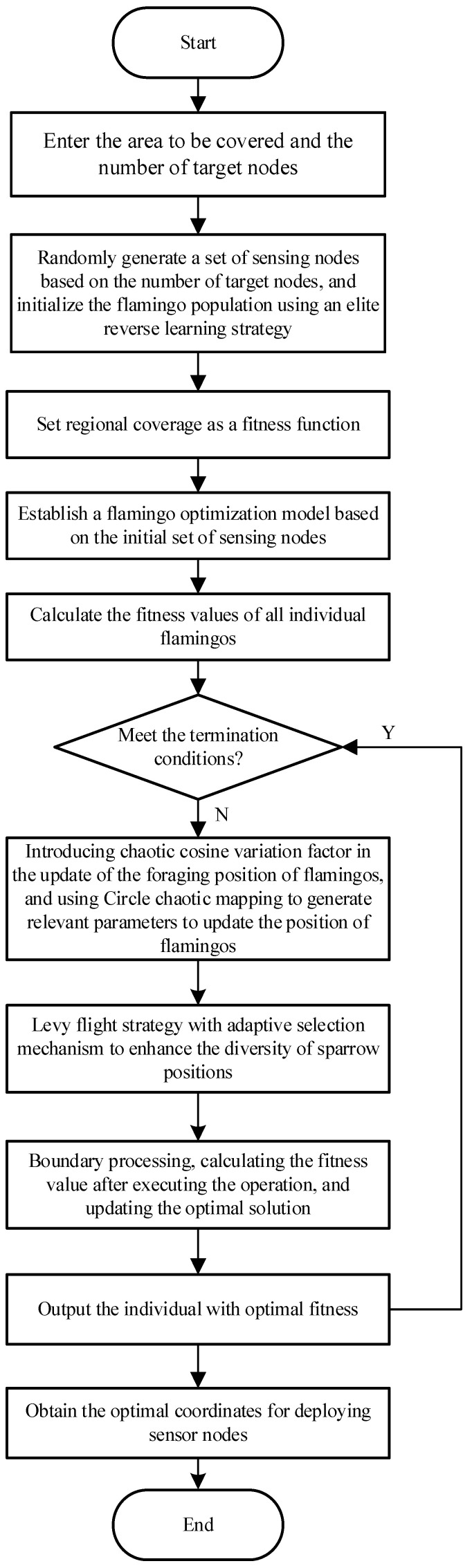
Flowchart of coverage optimization algorithm for wireless sensor networks based on MISFA algorithm.

The flowchart of coverage optimization algorithm for wireless sensor networks based on elite reverse learning strategy and flamingo search optimization algorithm is shown in Figure 2.
**Algorithm 1:** The MIFSAStart MIFSA. **Input**: Flamingo population size; Maximum number of iterations, *IferM_ax_*; The first part is the proportion of migrating flamingos, *MP_b_*. Output: The optimal fitness value, *f_g_*. The optimal solution, *X_best_*. 1. Using (12) update the flamingo’s location. 2. Rank the fitness values and find the current best individual, *X_best_*. 3. t ← 1. 4. while (t ≤ *lter_Max_*) do 5.    *R* ← *rand* [0,1]. 6.    *MP_r_* ← *R* × *P* × (1 − *MP_b_*). 7.    *MP_0_* ← *MP_b_*. 8.    *MP_t_* ← *P*-*MP_0_* 9.    for *i* <- 1 to *MP_b_do* 10.    for *j* < 1 to *n* //n is the dimension size 11.      Using (13) update the flamingo’s location; 12.      end for 13.    end for 14.    for i ← 1 + *MP_0_* to *MP_0_* + *MP_r_*
15.      for j ← 1 to n do 16.        Using (15) update the flamingo’s location; 17.      end for 18.    end for 19.    for *i* ← *MP_0_* + *MP_r_* + 1 to *P* 20.        for j ← 1 to *n*
21.         Using (16) update the flamingo’s location; 22.        end for 23.    end for 24.        Using (17) update the flamingo’s location; 25.    for i ← 1 to *P* //Boundary detection; 26.      *for j* ← *1 to d * 27.        if *x^t^_ij_* > *ub* then 28.         *x^t^_ij_* ← *ub* 29.      end if 30.      if *x^t^_ij_* < *lb* then 31.        *x^t^_ij_* ← *lb* 32.      end if 33.    end for 34.    end for 35.    Rank the fitness values and find the current best individual, *X_best_*. 36.    *t* ← *t* + 1 37. end while 38. return *f_g_* and *X_best_* End MIFSA.

## 5. Simulation Experimental Analysis

### 5.1. Experimental and Environmental Settings

In this paper, in addition to conducting experiments on the MIFSA, seven original algorithms are selected as comparison objects, including the Subtraction-Based Optimization Algorithm (SABO) [53], Beluga Whale Optimization Algorithm (BWO) [54], Kepler Optimization Algorithm (KOA) [55], Spider Wasp Optimization Algorithm (SWO) [56], Butterfly Optimization Algorithm (BOA) [57], Whale Optimization Algorithm (WOA) [58], and Flamingo Search Optimization Algorithm (FSA). They are compared through simulation experiments on the CEC2005 test set and two engineering applications. The superiority of the MIFSA algorithm is evaluated by the optimal value, average value, and standard deviation obtained from the function solutions. Detailed information regarding these functions is presented in Table 1.

In this comparative study, each algorithm was independently tested 30 times. The maximum number of iterations for each algorithm was set to 500, and the number of individuals in the population was set to 30. The other parameters of the comparative algorithms remained unchanged according to the settings in the original literature. The benchmark test functions used in the experiment covered unimodal functions, multimodal functions, and multimodal benchmark test functions with fixed dimensions. All numerical experiments were carried out in the Windows 10 operating system environment. The experimental platform was MATLAB R2023b.

### 5.2. Comparative Analysis Between MIFSA and Other Algorithms

**Figure 3:**
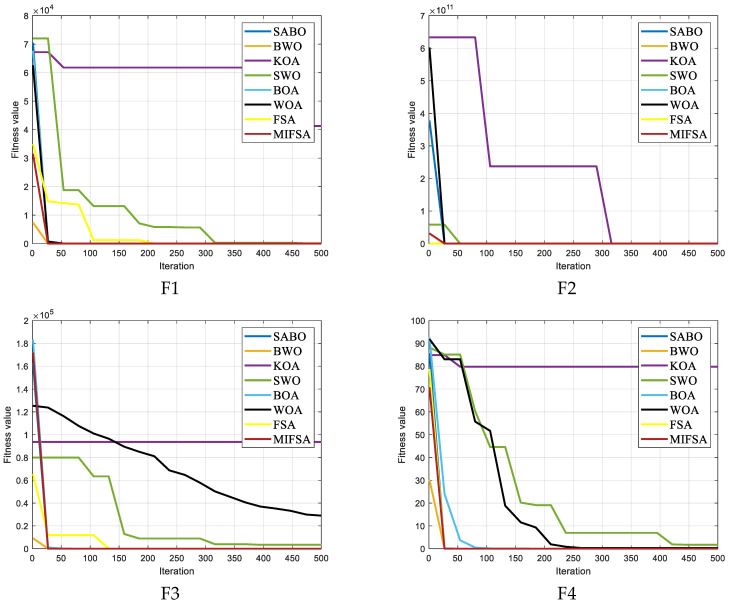

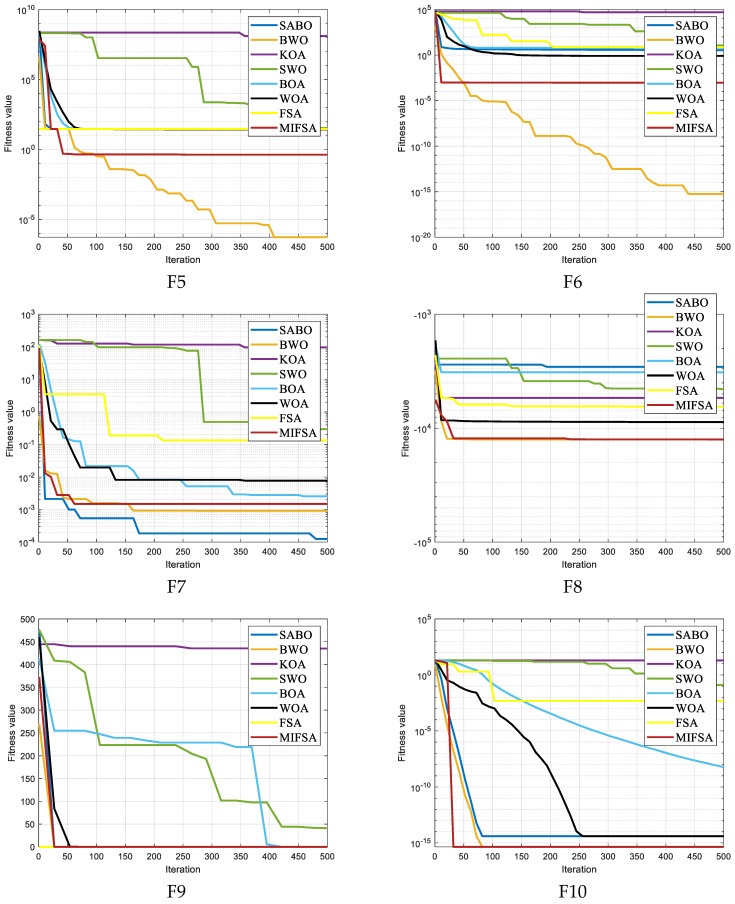

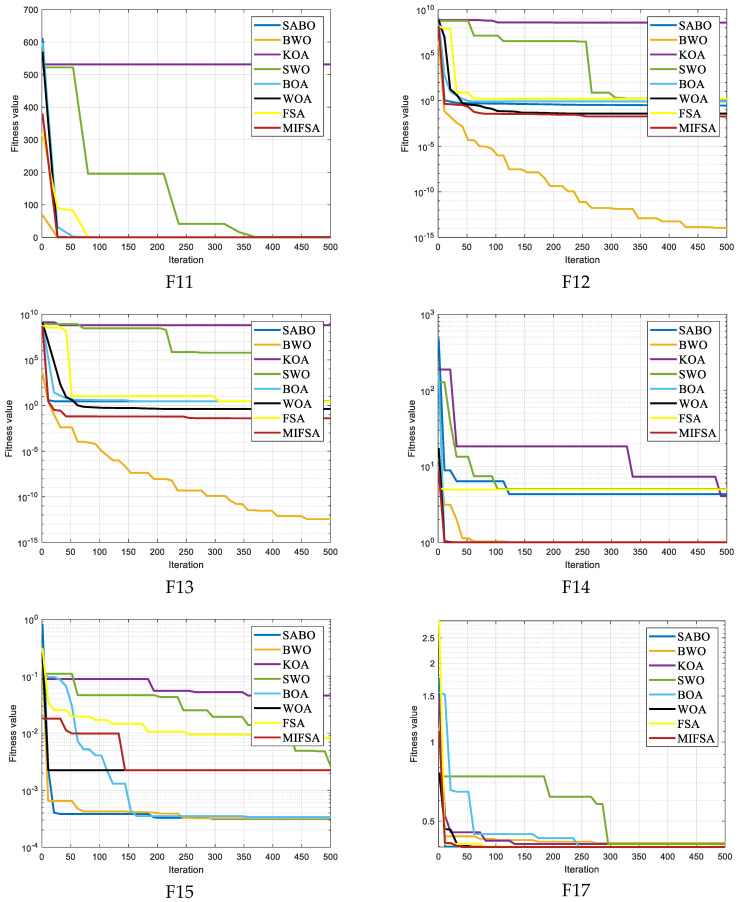

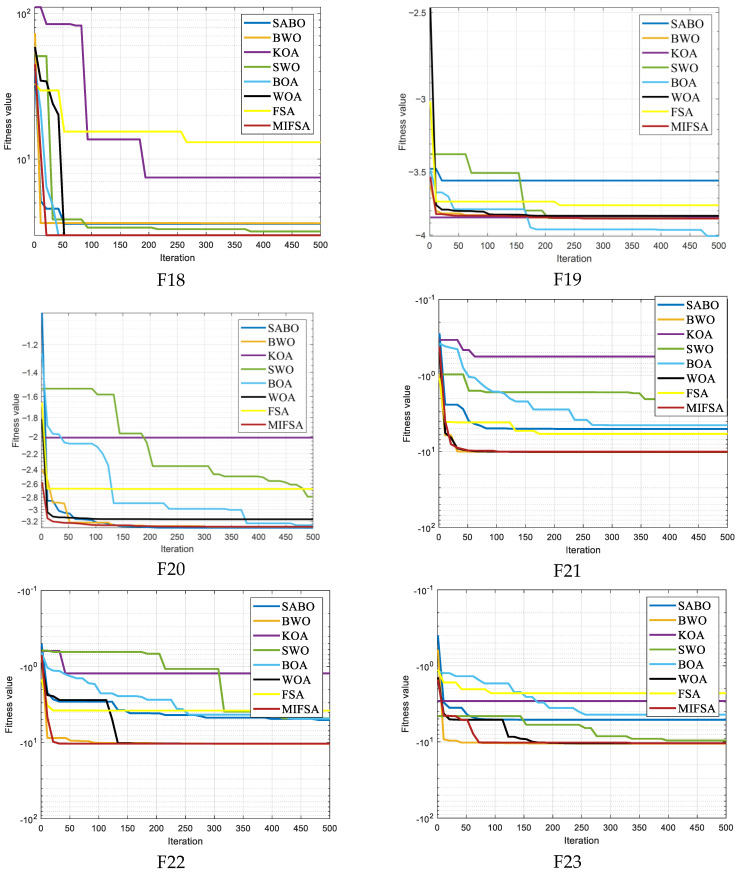
Iteration profile of different improved algorithms.

To verify the optimization performance of the MIFSA, it was compared with excellent algorithms in recent years. The CEC2005 test set functions were used in the experiment, and the test dimension (D = 20). Table 2 shows the experimental results of 23 independent runs, listing the optimal fitness value (min), average fitness value (avg), and standard deviation of the fitness value (std), where the bold data represents the best result. Figure 3 shows the average convergence curve during the iteration process.

As shown in the detailed data analysis of Table 1, when the MIFSA proposed in this study was tested against 22 standard test functions—covering both unimodal and multimodal optimization scenarios—it achieved the minimum optimal value in 15 of these functions, accounting for nearly 70% of the total test cases. Additionally, it secured the triple optimal value (typically referring to the best performance in terms of accuracy, stability, and convergence speed) in over half of the functions, a result that directly validates the effectiveness of the algorithm’s integrated improvement strategy (including elite opposition-based learning and chaotic cosine variation). Although in certain test functions with high complexity (e.g., F5, F14, F21, F22, and F23, which are characterized by narrow optimal regions or strong local optima interference), the optimal value of MIFSA is slightly lower than that of the BWO, its overall performance across all 22 functions—especially in terms of consistent optimal value output and adaptability to different function types—is more favorable, fully reflecting its greater practicality for real-world WSN coverage optimization tasks.

In Figure 2, which visualizes the convergence curve of F23 (a representative multimodal function with multiple local optima), both the MIFSA and the original FSA initially became trapped in a local optimum at the 10th iteration, showing similar stagnation trends at this stage. Nevertheless, the lens imaging opposition-based learning strategy integrated into MIFSA substantially strengthens the algorithm’s capability to break free from local optima: unlike FSA, which remained stuck in the same local optimum for subsequent iterations, the enhanced MIFSA was able to escape this local trap more rapidly. Specifically, MIFSA successfully broke away from the initial local optimum after 50 iterations; with just 10 additional iterations (by the 60th iteration), it reidentified the global optimal value and then immediately initiated further global exploration to prevent falling back into new local optima. From the 60th iteration to the end of the simulation (set to 200 iterations), the algorithm maintained stable convergence and did not get stuck in a local optimum again. At this late convergence stage, the chaotic cosine variation factor played a key role in improving the algorithm’s search efficiency: it fine-tuned the search step size to avoid redundant calculations while ensuring the algorithm stayed focused on the global optimal region, ultimately enabling it to obtain the theoretical optimal value of F23.

### 5.3. Further Comparative Experiments of the Algorithms (50D)

**Figure 4:**
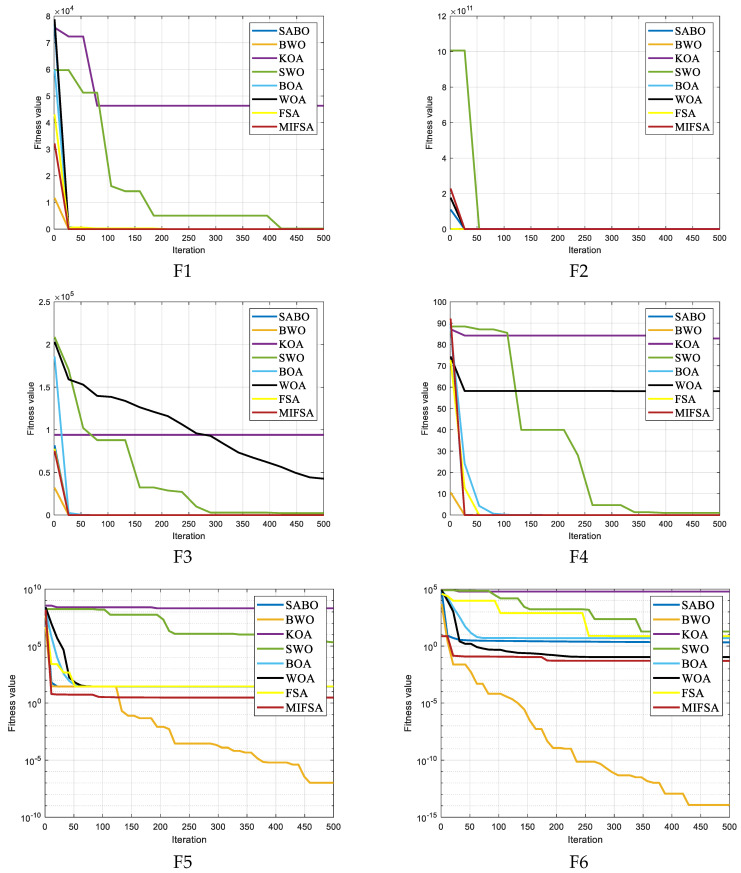

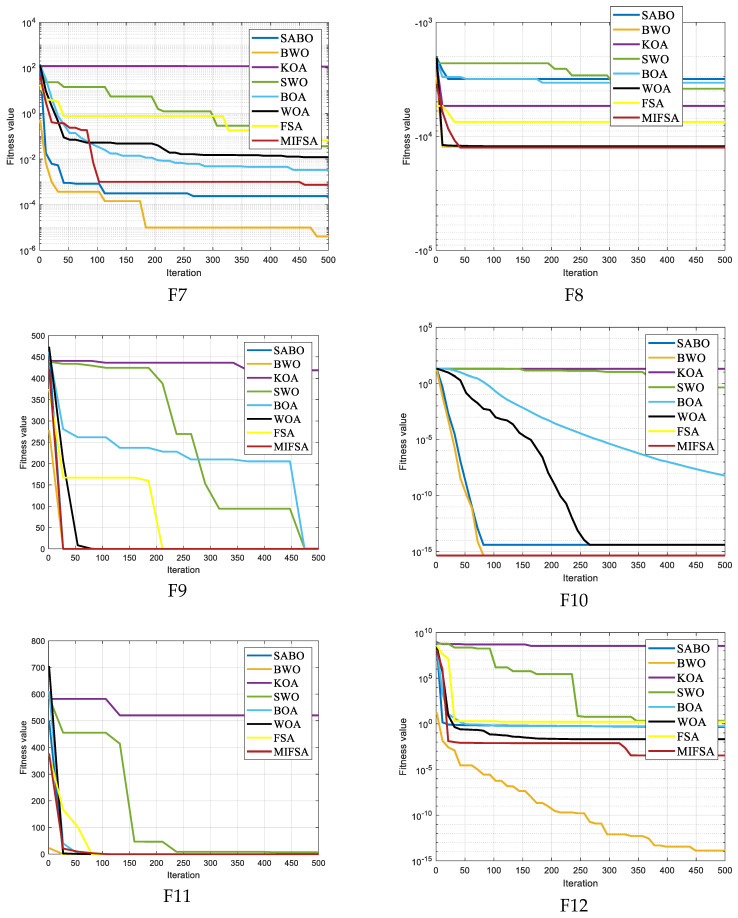

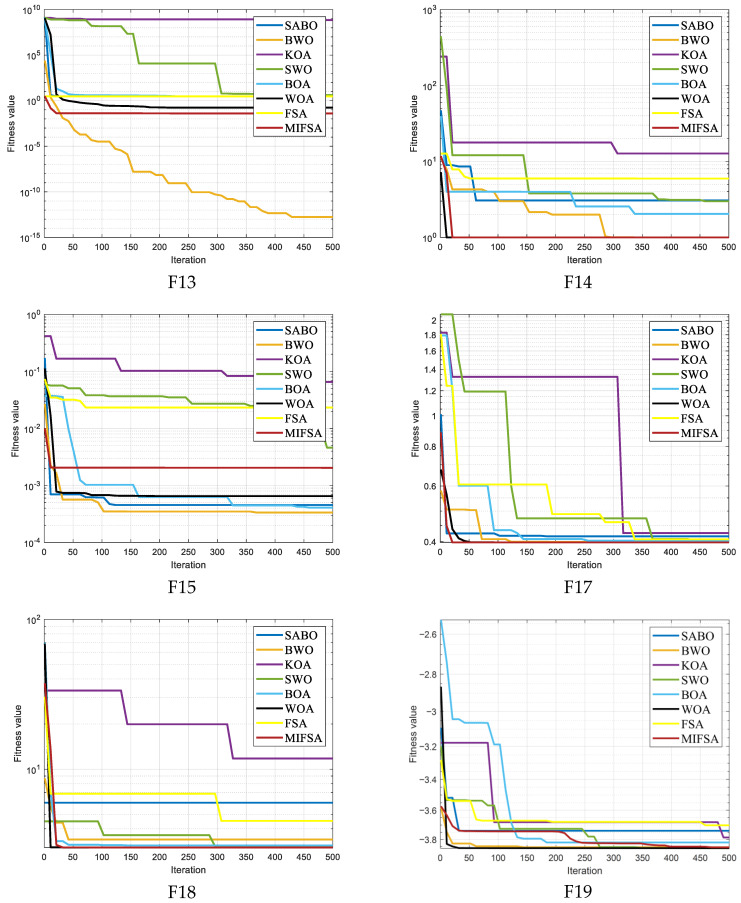

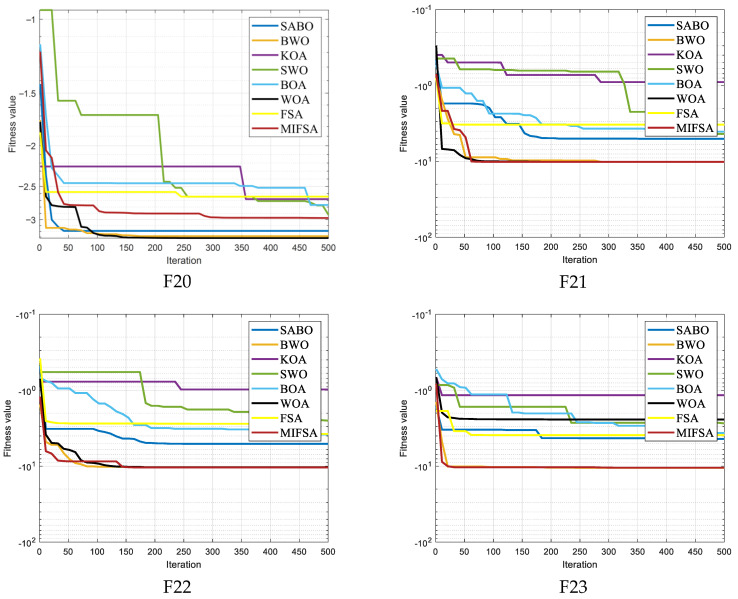
Iteration profile of different algorithms.

To further verify the feasibility and effectiveness of the proposed improved algorithm for high-dimensional problems, we applied the eight algorithms to solve problems in the CEC2005 test set with the test dimension D = 50. Table 3 records the optimal fitness value (min), average fitness value (avg), and standard deviation of the fitness value (std), where the bold data represents the best result. Figure 4 shows the average convergence curve during the iteration process.

As Table 2 demonstrates, except for the test functions F5, F6, F7, F12, F13, F15, and F20, the MIFSA can successfully pinpoint the optimal solution for all remaining test functions. Moreover, it achieves the triple optimal value—typically defined as the best performance in optimal value accuracy, result stability (reflected by standard deviation), and convergence speed—in more than half of the total test cases. This outcome clearly indicates that the enhanced Flamingo Search Algorithm (FSA) possesses high optimization precision, a key advantage for addressing complex WSN coverage optimization tasks. When focusing on unimodal test functions (F5, F6)—which evaluate an algorithm’s ability to converge to a single global optimum—and simple multimodal test functions (F12, F13)—which introduce multiple local optima but with relatively clear search landscapes—the optimization precision of MIFSA is slightly lower than that of the BWO. However, even in these cases, the gap is negligible: although MIFSA does not achieve the minimum standard deviation or optimal value for F5, F6, and F8, the differences between its performance and the best-performing algorithm are not statistically significant. For the combined functions spanning F14 to F23—complex test cases that place higher demands on an algorithm’s optimization precision and efficiency due to their hybrid search landscapes (integrating unimodal, multimodal, and noisy sub-regions)—MIFSA exhibits excellent solution precision. It basically secures the best optimal value for most of these functions and further attains the triple optimal value in 7 out of this 10-function subset. Only in F15 and F20 does MIFSA fail to rank first in terms of triple values among all compared algorithms. Even so, this result still reinforces the improved algorithm’s strong solution precision and stability, as it maintains competitive performance across the majority of high-difficulty test cases.

The MIFSA also performs exceptionally well in high-dimensional scenarios: it maintains consistent effectiveness in both 20-dimensional and 50-dimensional spaces when tackling complex multi-dimensional combined functions and hybrid functions. Notably, it can overcome the constraints of local optima in the vast majority of these complex functions and ultimately locate the global optimal value—a critical capability for scaling to large-scale WSN deployments (where node deployment involves dozens of coordinate variables). This high-dimensional optimization performance is more intuitively illustrated in the iteration curve diagram of Figure 3, which tracks the algorithm’s convergence trend across dimensions. As observed in Figure 4, while the improved MIFSA may still become trapped in local optima for some functions during early iterations, the integration of the cosine variation factor and stage-controlled step size strategy enables it to effectively break free from local optimum limitations in the later stages of iteration. Taking test functions F7, F21, and F22 as typical examples: the traditional FSA remains stuck in local optima indefinitely after encountering them, unable to make further progress toward the global optimum. In contrast, when processing these same functions, the improved MIFSA exhibits a strong ability to escape local traps in the later phase of its convergence curve—this escape is manifested as a sudden drop in the objective function value (toward the global optimum) after the 50th to 100th iteration, thereby significantly boosting convergence precision. Overall, the stability of the MIFSA outperforms that of the original FSA: this advantage is particularly pronounced in the testing of complex functions (e.g., F14–F23 and high-dimensional cases), where MIFSA delivers far more outstanding and consistent performance. By synthesizing the above experimental results, it can be concluded that the MIFSA possesses superior convergence efficiency (faster arrival at optimal values) and robustness (consistent performance across function types and dimensions)—two core strengths for practical WSN coverage optimization applications.

### 5.4. WSNs Coverage Optimization Simulation Experiment and Analysis

**Figure 5:**
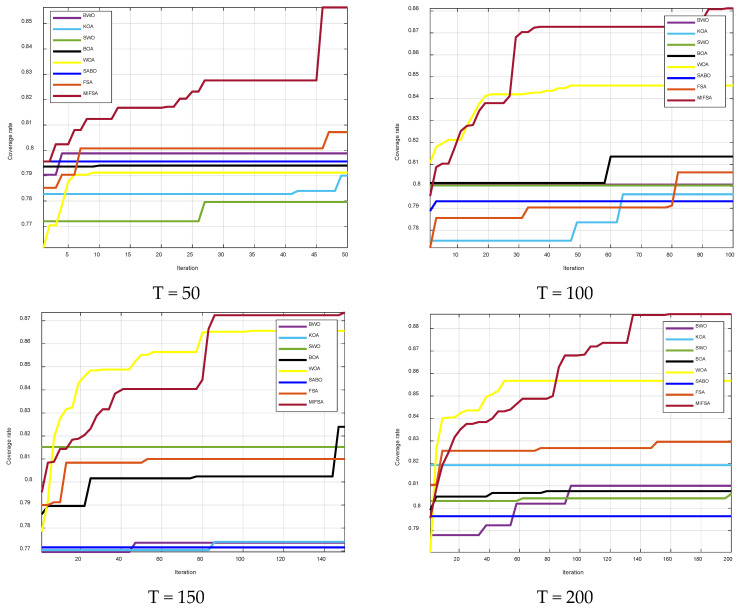
Algorithm Comparison Iteration Diagram.

To verify the effectiveness and superiority of the MIFSA proposed in this paper in improving the WSN coverage rate, a simulation experiment was carried out using MATLAB 2023b. The experiment conducted a two-dimensional node deployment coverage analysis and compared the MIFSA with other optimization algorithms. The experimental parameter settings were as follows: the monitoring area was 50 × 50, the number of nodes *N* = 30, and the sensing radius *r* = 6. This scenario belonged to the WSNs deployment within the normal area range and was closer to the real-world network scenario. The algorithm parameters were set as the number of population individuals being 50, and the maximum number of iterations T being 50, 100, 150, and 200, respectively. Figure 5 shows the iteration curves after optimizing with the MIFSA, FSA algorithm, and 6 other optimization algorithms. The operation results of the 8-algorithm optimization are shown in Table 4.

As can be seen from Figure 5, the BOA, BWO, and SABO seriously fell into the local-optimum problem during the iteration process, resulting in low convergence accuracy. Although the WOA had a fast convergence speed in the initial stage of iteration, it encountered a serious local-optimum problem at 50 iterations and failed to effectively jump out of the local optimum. The FSA algorithm had a slow convergence speed in the initial stage and focused more on global search, but finally obtained a relatively good result.

The improved algorithm MIFSA proposed in this paper had a slow convergence speed in the initial stage due to the introduction of the cosine variation factor. However, after 25 iterations, the adjustment of the cosine variation factor enabled the algorithm to enter the in-depth development stage, and it found the optimal value in the subsequent 95 iterations. The introduction of the chaotic cosine variation factor made the convergence speed of MIFSA much faster than that of the original algorithm in the later stage of iteration, showing superiority. To comprehensively evaluate the algorithm performance, this section of the experiment conducted comparative experiments with the maximum number of iterations of 50, 100, 150, and 200. The results showed that with the increase in the maximum number of iterations, the cosine variation factor of MIFSA played a better role: it not only enhanced the global optimization ability in the early stage and accelerated the convergence speed in the early stage but also maintained a significant convergence speed in the in-depth development stage.

**Figure 6:**
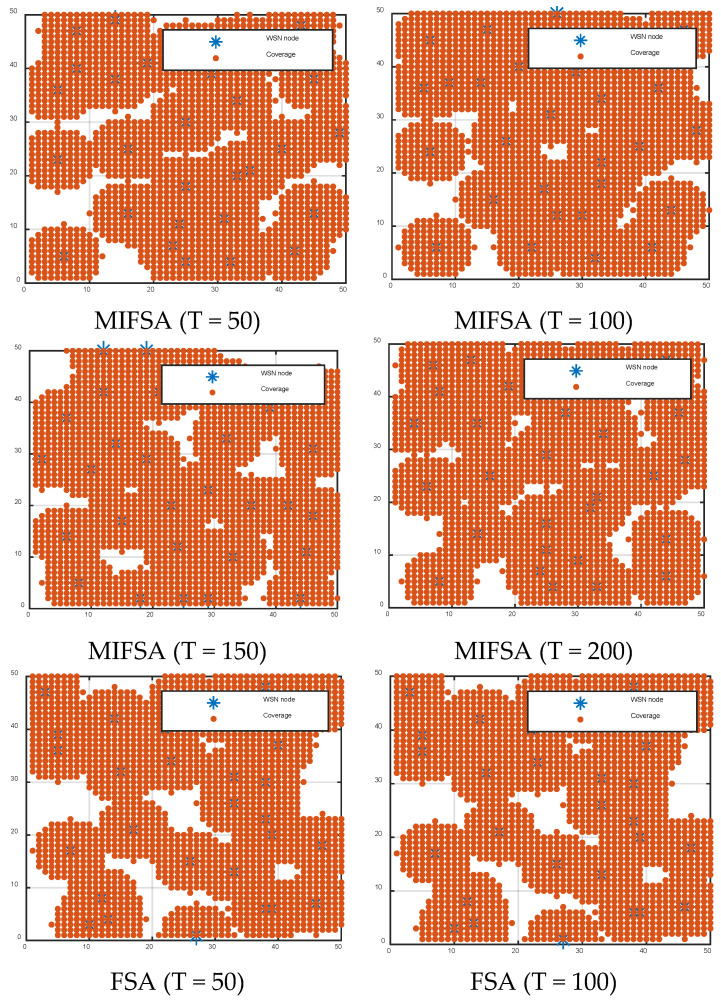

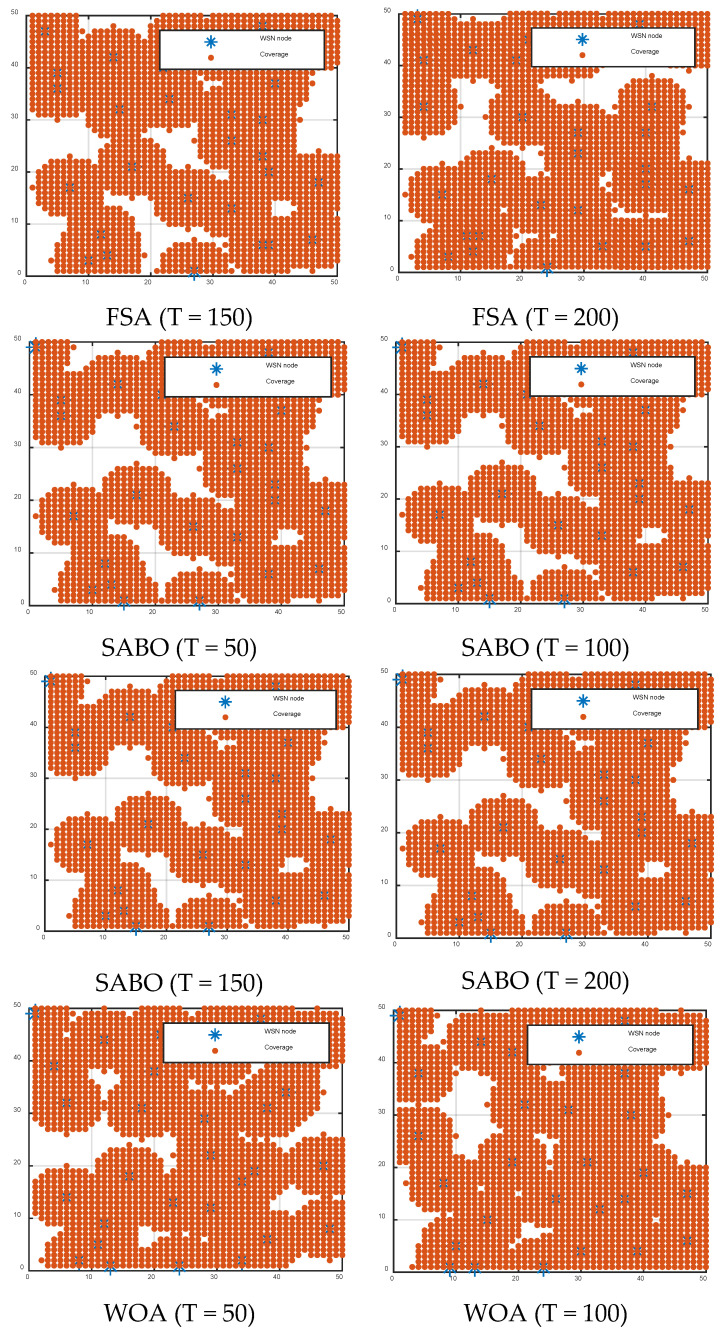

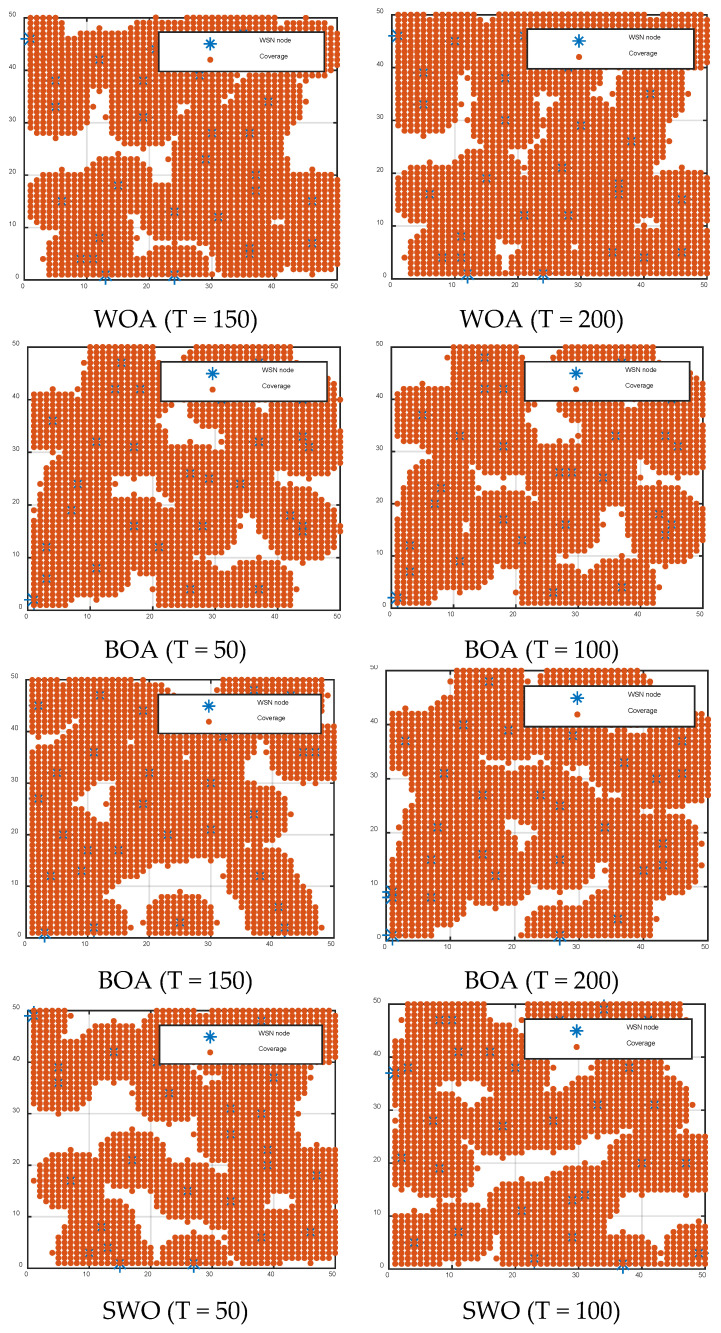

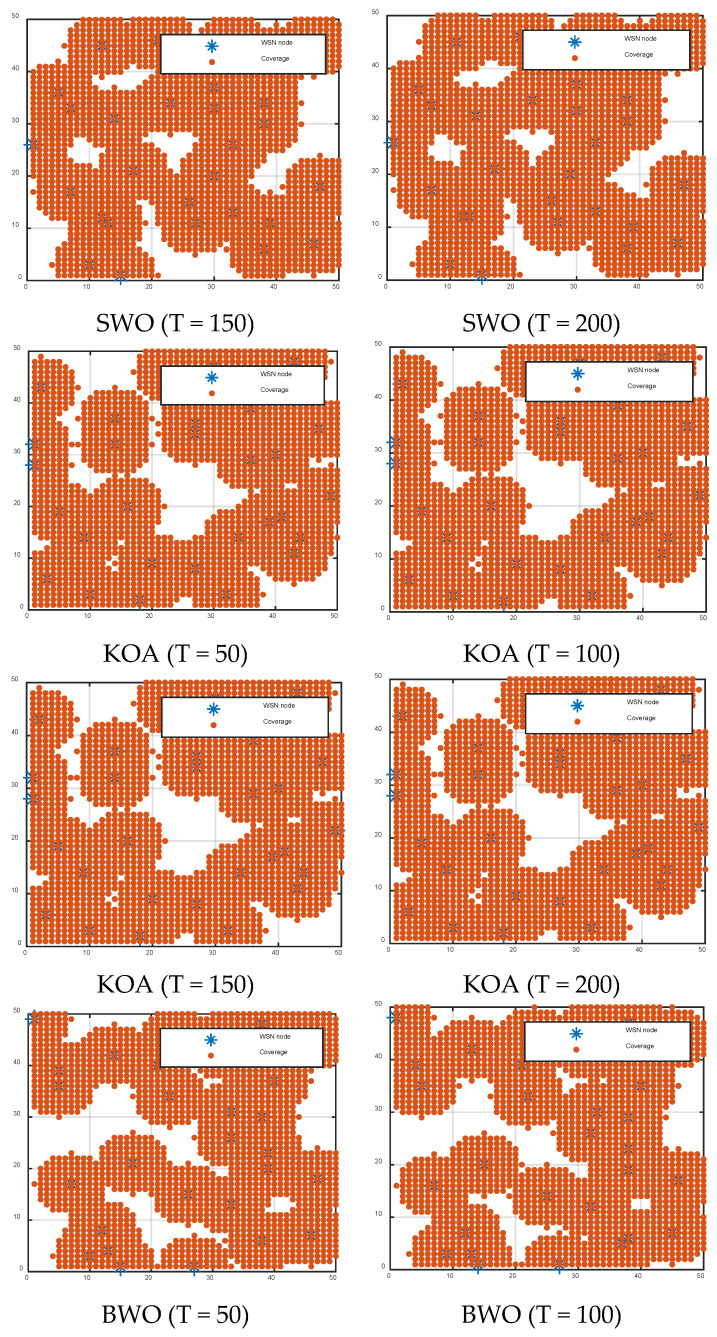

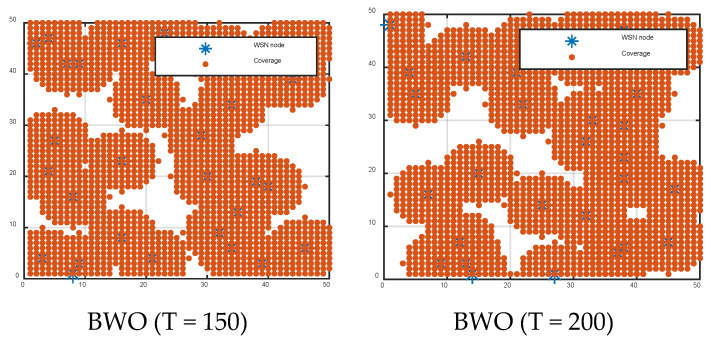
WSNs coverage effect diagrams of different algorithms.

From the analysis of Table 3, it can be seen that the coverage rates of the MIFSA in 50, 100, 150, and 200 iterations increased by 4.92%, 7.48%, 7.35%, and 5.68%, respectively, compared with the original FSA. Figure 6 further clearly shows the practical performance of this data.

At the same time, we conducted a sensor node deployment coverage effect experiment. The experimental parameter settings were: the monitoring area was 50 × 50, the number of nodes *N* = 30, and the sensing radius *r* = 6. The algorithm parameters were set as the number of population individuals being 50, and the maximum number of iterations T being 50, 100, 150, and 200, respectively. Figure 5 shows the node coverage effects optimized by the 8 algorithms.

In the wireless sensor network (WSN) coverage diagrams in Figure 6, the node deployment effect of the improved MIFSA after 50, 100, 150, and 200 iterations was significantly improved compared with that of the FSA, and the distribution of sensor nodes was more uniform. After 200-iteration optimization, it almost achieved full coverage, and the small uncovered areas were relatively close. However, the FSA had a large coverage blind area and a high repetition rate after 100 iterations, and there were still many coverage blind areas after 200 iterations. The other comparative algorithms also had the same shortcomings. From the coverage rate and the coverage optimization diagram, it can be seen that the improved MIFSA can effectively improve the network coverage rate.

## 6. Conclusions

This research proposes an enhanced Flamingo Search Algorithm (MIFSA) to address the coverage optimization challenge in two-dimensional wireless sensor network node deployment, with its effectiveness validated via simulation experiments. Compared to the original Flamingo Search Algorithm (FSA), MIFSA incorporates targeted improvements to overcome key coverage-related limitations: First, during initialization, it integrates an elite opposition-based learning method—by generating opposite solutions and selecting elite entities, this technique expands the algorithm’s search range, enhances global search capability, and reduces the risk of falling into local optima (a major barrier to achieving full coverage). Second, a phased step-size control strategy (via a warning value-based spiral search mechanism) is introduced to balance search scope expansion and gradual convergence toward optimal node positions, directly addressing the trade-off between coverage breadth and deployment precision. Third, a nonlinear attenuation factor μ is added to boost search accuracy and convergence rate: it enables broad regional exploration in early iterations and focused optimization of high-potential areas in mid-to-late stages. Finally, a chaotic cosine variation factor is integrated to balance exploration and exploitation across iterations, promoting in-depth exploration of uncharted regions and further lowering the probability of local minima trapping—an issue that often leads to incomplete coverage. Quantitatively, MIFSA achieves coverage rate increases of 7.48% (after 100 iterations) and 5.68% (after 200 iterations) compared to the original FSA, clearly demonstrating its effectiveness in advancing WSN coverage performance and addressing core coverage optimization challenges.

Despite these advances, this study has notable limitations related to coverage optimization applicability: First, MIFSA is currently restricted to two-dimensional planar WSN environments, failing to address coverage challenges in three-dimensional (3D) scenarios (e.g., aerial-ground integrated WSNs or underground monitoring networks) where node deployment and coverage calculation are more complex. Second, the algorithm does not account for dynamic coverage changes caused by node mobility or energy depletion—factors that frequently lead to coverage degradation in real-world WSN operations. Third, while MIFSA improves coverage rate, its performance in large-scale WSNs (with hundreds or thousands of nodes) has not been fully validated, and computational efficiency may decline with increasing network size, limiting its practical application in large-area monitoring. Corresponding future research directions include: (1) Extending MIFSA to 3D WSNs by modifying coverage models to account for spatial depth and adjusting search mechanisms to adapt to 3D node deployment constraints; (2) Integrating dynamic node state awareness (e.g., real-time energy levels, mobility patterns) into MIFSA to enable adaptive coverage optimization and maintain long-term coverage stability; (3) Optimizing the algorithm’s computational complexity (e.g., via parallel computing or heuristic simplification) to enhance its scalability for large-scale WSNs, while validating its performance in real-world engineering applications to improve practical utility.

## Figures and Tables

**Table 1:** CEC2005 Test Functions.

Function	Equation	Dimension	Bounds	Optimum
F1	$\sum_{i=1}^{d}x_{i}^{2}$	30	[−100,100]	0
F2	$\sum_{i=1}^{d}\left|x_{i}\right|+\prod_{i=1}^{d}\left|x_{i}\right|$	30	[−10,100]	0
F3	${\sum_{i=1}^{d}\left(\sum_{j=1}^{i}x_{j}\right)}^{2}$	30	[−100,100]	0
F4	$\max\left\{\left|x_{i}\right|,1\leq i\leq d\right\}$	30	[−100,100]	0
F5	$\sum_{i=1}^{d-1}\left[100{\left(x_{i+1}-x_{i}^{2}\right)}^{2}+{\left(x_{i}-1\right)}^{2}\right]$	30	[−10,100]	0
F6	$\sum_{i=1}^{d}{\left(\left|x_{i}+0.5\right|\right)}^{2}$	30	[−100,100]	0
F7	$\sum_{i=1}^{d}ix_{i}^{4}+rand(0,1)$	30	[−1.28,1.28]	0
F8	$\sum_{i=1}^{d}\left|x_{i}\sin(x_{i})+0.1x_{i}\right|$	30	[−10,100]	0
F9	$10d+\sum_{i=1}^{d}\left[x_{i}^{2}-10\cos(2\pi x_{i})\right]$	30	[−100,100]	0
F10	$-20\exp\left(-0.2\sqrt{\frac{1}{d}\sum_{i=1}^{d}x^{2}}\right)-\exp\left(\frac{1}{d}\sum_{i=1}^{d}\cos(2\pi x_{i})\right)+20+\exp(1)$	30	[−5.12,5.12]	0
F11	$\frac{1}{4000}\sum_{i=1}^{d}x_{i}^{2}-\prod_{i=1}^{d}\cos\frac{x_{i}}{\sqrt{i}}+1$	30	[−32,32]	0
F12	$\begin{array}{l} F12=\frac{\pi }{d}\left\{10\sin(\pi y_{1})+\sum_{i=1}^{d-1}{\left(y_{i}-1\right)}^{2}[1+10\sin^{2}(\pi y_{i+1})]+{\left(y_{n}-1\right)}^{2}\right\}\\ \;\;\;\;+\sum_{i=1}^{d}u(x_{i},10,100,4)\\ y_{i}=1+\frac{x_{i}+1}{4}u\left(x_{i},a,k,m\right)=\left\{\begin{array}{c} k{\left(x_{i}-a\right)}^{m},x_{i}>a\\ 0,-a<x_{i}<a\\ k{\left(-x_{i}-a\right)}^{m},x_{i}<-a \end{array}\right. \end{array}$	30	[−600,600]	0
F13	$\begin{array}{l} 0.1\{\sin^{2}(3\pi x_{1})+\sum_{i=1}^{d-1}{\left(x_{i}-1\right)}^{2}[1+\sin^{2}(3\pi x_{i}+1)]\\ +{\left(x_{n}-1\right)}^{2}[1+\sin^{2}(2\pi x_{n})]\}+\sum_{i=1}^{d}u(x_{i},5,100,4) \end{array}$	30	[−50,50]	0
F14	${\left(\frac{1}{500}+\sum_{j=1}^{25}\frac{1}{j+\sum_{i=1}^{2}{\left(x_{i}-a_{ij}\right)}^{6}}\right)}^{-1}$	2	[−65,65]	0
F15	$\sum_{i=1}^{11}{\left[a_{i}-\frac{x_{i}(b_{i}^{2}+b_{i}x_{2})}{b_{i}^{2}+b_{i}x_{3}+x_{4}}\right]}^{2}$	4	[−5,5]	0.0003
F16	$4x_{1}^{2}-2.1x_{1}^{4}+\frac{1}{3}x_{1}^{6}+x_{1}x_{2}-4x_{2}^{2}+4x_{2}^{4}$	2	[−5,5]	−1.0316
F17	${\left(x_{2}-\frac{5.1}{4\pi^{2}}x_{1}^{2}+\frac{5}{\pi }x_{1}-6\right)}^{2}+10(1-\frac{1}{8\pi })\cos x_{1}+10$	2	[−5,10]	0.3978
F18	$\begin{array}{l} {}[1+{\left(x_{1}+x_{2}+1\right)}^{2}(19-14x_{1}+3x_{1}^{2}-14x_{2}+\\ 6x_{1}x_{2}+3x_{2}^{2})]\times [30+{\left(2x_{1}-3x_{2}\right)}^{2}\times (18-32x_{1}\\ +12x_{1}^{2}+48x_{2}-36x_{1}x_{2}+27x_{2}^{2})] \end{array}$	2	[−2,2]	3
F19	$-\sum_{i=1}^{4}c_{i}\exp\left(-\sum_{j=1}^{3}a_{ij}{\left(x_{j}-p_{ij}\right)}^{2}\right)$	3	[1,3]	−3.86
F20	$-\sum_{i=1}^{4}c_{i}\exp\left(-\sum_{j=1}^{6}a_{ij}{\left(x_{j}-p_{ij}\right)}^{2}\right)$	6	[0,1]	−3.32
F21	$-\sum_{i=1}^{5}{\left[(X-a_{i}){\left(X-a_{i}\right)}^{T}+c_{i}\right]}^{-1}$	4	[0,10]	−10.1532
F22	$-\sum_{i=1}^{7}{\left[(X-a_{i}){\left(X-a_{i}\right)}^{T}+c_{i}\right]}^{-1}$	4	[0,10]	−10.4028
F23	$-\sum_{i=1}^{10}{\left[(X-a_{i}){\left(X-a_{i}\right)}^{T}+c_{i}\right]}^{-1}$	4	[0,10]	−10.5363

**Table 2:** Solution results of different swarm intelligence algorithms (D = 20).

Function		MIFSA	FSA	WOA	BOA	SWO	KOA	BWO	SABO
F1	min	0	0	3.96 × 10^−85^	1.12 × 10^−11^	1.82 × 10^−2^	4.05 × 10^4^	6.23 × 10^−273^	3.87 × 10^−202^
F1	std	0	2.59	2.54 × 10^−75^	9.31 × 10^−13^	8.4 × 10^2^	6.73 × 10^3^	0	0
F1	avg	0	5.11 × 10^−1^	1.36 × 10^−75^	1.31 × 10^−11^	3.83 × 10^2^	5.46 × 10^4^	4.14 × 10^−259^	1.03 × 10^−196^
F2	min	0	0	7.89 × 10^−59^	2.1 × 10^−9^	1.69 × 10^−2^	2.54 × 10^5^	3.91 × 10^−138^	2.00 × 10^−113^
F2	std	0	3.77 × 10^−2^	1.13 × 10^−51^	1.31 × 10^−9^	4.08	4.64 × 10^10^	2.21 × 10^−130^	8.59 × 10^−111^
F2	avg	0	1.05 × 10^−2^	4.35 × 10^−52^	4.53 × 10^−9^	2.47	2.00 × 10^10^	4.53 × 10^−131^	4.30 × 10^−111^
F3	min	0	0	1.35 × 10^4^	1.12 × 10^−11^	2.54 × 10^−1^	5.82 × 10^4^	1.13 × 10^−257^	3.90 × 10^−95^
F3	std	0	1.22 × 10^−2^	1.65 × 10^4^	8.98 × 10^−13^	6.83 × 10^3^	1.50 × 10^4^	0	1.02 × 10^−26^
F3	avg	0	2.65 × 10^−3^	4.46 × 10^4^	1.29 × 10^−11^	2.79 × 10^3^	8.52 × 10^4^	5.08 × 10^−239^	1.87 × 10^−27^
F4	min	0	0	7.77 × 10^−1^	5.54 × 10^−9^	4.17 × 10^−2^	7.31 × 10^1^	9.52 × 10^−133^	1.31 × 10^−78^
F4	std	0	1.20 × 10^−2^	2.51 × 10^1^	2.93 × 10^−10^	6.53	3.1	5.45 × 10^−126^	5.81 × 10^−77^
F4	avg	0	2.50 × 10^−3^	4.44 × 10^1^	6.08 × 10^−9^	4.85	8.17 × 10^1^	1.09 × 10^−126^	3.57 × 10^−77^
F5	min	9.15 × 10^−6^	2.88 × 10^1^	2.73 × 10^1^	2.89 × 10^1^	2.9 × 10^1^	1.25 × 10^8^	7.51 × 10^−9^	2.79 × 10^1^
F5	std	3.82	9.73	4.86 × 10^−1^	2.61 × 10^−2^	4.29 × 10^5^	2.66 × 10^7^	1.14 × 10^−5^	3.04 × 10^−1^
F5	avg	2.75	3.16 × 10^1^	2.82 × 10^1^	2.89 × 10^1^	8.4 × 10^4^	1.87 × 10^8^	3.96 × 10^−6^	2.85 × 10^1^
F6	min	1.48 × 10^−3^	1.39	7.44 × 10^−2^	4.92	6.21	4.54 × 10^4^	2.28 × 10^−16^	1.78
F6	std	4.28 × 10^−1^	2.39	2.31 × 10^−1^	6.06 × 10^−1^	1.93 × 10^2^	5.47 × 10^3^	3.74 × 10^−14^	4.84 × 10^−1^
F6	avg	3.02 × 10^−1^	6.69	3.99 × 10^−1^	5.95	9.5 × 10^1^	5.64 × 10^4^	2.35 × 10^−14^	2.64
F7	min	1.07 × 10^−4^	4.13 × 10^−3^	1.13 × 10^−4^	2.46 × 10^−4^	6.89 × 10^−3^	7.02 × 10^1^	1.55 × 10^−6^	1.23 × 10^−5^
F7	std	5.08 × 10^−3^	1.03 × 10^−1^	3.78 × 10^−3^	5.38 × 10^−4^	7.05 × 10^−2^	1.13 × 10^1^	8.13 × 10^−5^	1.06 × 10^−4^
F7	avg	4.53 × 10^−3^	9.18 × 10^−2^	3.32 × 10^−3^	1.27 × 10^−3^	7.05 × 10^−2^	8.93 × 10^1^	1.09 × 10^−4^	1.41 × 10^−4^
F8	min	−1.26 × 10^4^	−1.08 × 10^4^	−1.26 × 10^4^	−3.07 × 10^3^	−4.95 × 10^3^	−5.42 × 10^3^	−1.26 × 10^4^	−4.03 × 10^3^
F8	std	6.64 × 10^2^	9.91 × 10^2^	1.99 × 10^3^	4.42 × 10^2^	4.06 × 10^2^	1.85 × 10^−12^	1.16 × 10−8	3.25 × 10^2^
F8	avg	−1.22 × 10^4^	−6.98 × 10^3^	−1.01 × 10^4^	−3.81 × 10^3^	−4.25 × 10^3^	−5.42 × 10^3^	−1.26 × 10^4^	−3.21 × 10^3^
F9	min	0	0	0	0	5.07 × 10^−3^	3.57 × 10^2^	0	0
F9	std	0	1.03 × 10^−2^	1.04 × 10^−14^	7.55 × 10^1^	5.25 × 10^1^	2.05 × 10^1^	0	0
F9	avg	0	2.88 × 10^−3^	1.89 × 10^−15^	3.32 × 10^1^	3.9 × 10^1^	3.98 × 10^2^	0	0
F10	min	4.44 × 10^−16^	4.44 × 10^−16^	4.44 × 10^−16^	5.28 × 10^−9^	6.9 × 10^−3^	1.98 × 10^1^	4.44 × 10^−16^	4.00 × 10^−15^
F10	Std	0	4.46 × 10^−2^	2.42 × 10^−15^	5.14 × 10^−10^	3.24	3.70 × 10^−2^	0	0
F10	avg	4.44 × 10^−16^	1.11 × 10^−2^	4.47 × 10^−15^	6.15 × 10^−9^	2.36	2 × 10^1^	4.44 × 10^−16^	4.00 × 10^−15^
F11	min	0	0	0	1.44 × 10^−12^	2.06 × 10^−4^	4.37 × 10^2^	0	0
F11	std	0	1.72 × 10^−2^	3.38 × 10^−2^	2.00 × 10^−12^	1.37	4.57 × 10^1^	0	0
F11	avg	0	4.18 × 10^−3^	6.17 × 10^−3^	4.31 × 10^−12^	1.6	5.26 × 10^2^	0	0
F12	min	1.88 × 10^−5^	1.91 × 10^−1^	6.43 × 10^−3^	2.7 × 10^−1^	5.96 × 10^−1^	2.15 × 10^8^	2.19 × 10^−16^	8.18 × 10^−2^
F12	std	2.51 × 10^−2^	6.39 × 10^−1^	1.35 × 10^−2^	1.59 × 10^−1^	4.8 × 10^2^	8.63 × 10^7^	5.32 × 10^−14^	2.30 × 10^−1^
F12	avg	1.59 × 10^−2^	1.19	2.54 × 10^−2^	5.95 × 10^−1^	9.02 × 10^1^	3.7 × 10^8^	2.64 × 10^−14^	2.74 × 10^−1^
F13	min	1.79 × 10^−4^	6.04 × 10^−1^	1.97 × 10^−1^	2.41	3	3.96 × 10^8^	1.67 × 10^−15^	1.66
F13	std	1.79 × 10^−1^	9.3 × 10^−1^	2.77 × 10^−1^	1.9 × 10^−1^	3.83 × 10^6^	1.47 × 10^8^	7.14 × 10^−13^	4.45 × 10^−1^
F13	avg	1.13 × 10^−1^	2.05	5.38 × 10^−1^	2.88	7.00 × 10^5^	7.99 × 10^8^	3.58 × 10^−13^	2.76
F14	min	9.98 × 10^−1^	1.01	9.98 × 10^−1^	9.98 × 10^−1^	9.98 × 10^−1^	2.12	9.98 × 10^−1^	9.98 × 10^−1^
F14	std	1.46	3.32	3.26	4.79 × 10^−1^	3.32	5.48	1.81 × 10^−1^	2.38
F14	avg	2.55	4.62	3.26	1.23	4.48	9.89	1.03	3.05
F15	min	4.25 × 10^−4^	2.25 × 10^−3^	3.11 × 10^−4^	3.27 × 10^−4^	7.05 × 10^−4^	2.73 × 10^−3^	3.08 × 10^−4^	3.21 × 10^−4^
F15	std	8.03 × 10^−3^	9.02 × 10^−3^	5.92 × 10^−4^	3.22 × 10^−4^	9.21 × 10^−3^	2.02 × 10^−2^	1.23 × 10^−4^	4.24 × 10^−3^
F15	avg	4.91 × 10^−3^	1.08 × 10^−2^	8.42 × 10^−4^	5.11 × 10^−4^	8.19 × 10^−3^	3.12 × 10^−2^	4.13 × 10^−4^	1.36 × 10^−3^
F17	min	3.98 × 10^−1^	3.98 × 10^−1^	3.98 × 10^−1^	3.98 × 10^−1^	3.98 × 10^−1^	4.05 × 10^−1^	3.98 × 10^−1^	3.98 × 10^−1^
F17	std	1.03 × 10^−6^	1.08 × 10^−1^	7 × 10^−6^	1.99 × 10^−3^	4 × 10^−2^	1.46 × 10^−1^	4.9 × 10^−3^	8.34 × 10^−2^
F17	avg	3.98 × 10^−1^	4.67 × 10^−1^	3.98 × 10^−1^	4.00 × 10^−1^	4.35 × 10^−1^	5.61 × 10^−1^	4.01 × 10^−1^	4.43 × 10^−1^
F18	min	3	3.01	3	3	3	3.24	3	3
F18	std	2.10 × 10^−6^	1.67	1.20 × 10^−4^	1.66 × 10^−1^	9.09 × 10^−1^	5.69	7.49 × 10^−1^	7.22
F18	avg	3	4.26	3	3.12	3.4	9.27	3.64	7.33
F19	min	−3.86	−3.86	−3.86	−3.82	−3.86	−3.85	−3.86	−3.86
F19	std	5.45 × 10^−2^	1.38 × 10^−1^	8.12 × 10^−3^	1.56 × 10^−1^	6.96 × 10^−3^	1.03 × 10^−1^	2.88 × 10^−3^	2.51 × 10^−1^
F19	avg	−3.84	−3.77	−3.86	−3.96	−3.86	−3.72	−3.86	−3.55
F20	min	−3.32	−3.05	−3.32	−2.58	−3.30	−3.08	−3.32	−3.32
F20	std	1.06 × 10^−1^	3.94 × 10^−1^	2.08 × 10^−1^	5.15 × 10^1^	1.27 × 10^−1^	3.01 × 10^−1^	3.75 × 10^−2^	1.32 × 10^−1^
F20	avg	−3.17	−2.54	−3.19	−1.59 × 10^1^	−3.07	−2.38	−3.28	−3.24
F21	min	−1.02 × 10^1^	−8.62	−1.02 × 10^1^	−4.87	−9.13	−1.94	−1.02 × 10^1^	−7
F21	std	4.22 × 10^−1^	1.43	2.81	2.07 × 10^−1^	2.17	4.28 × 10^−1^	4.07 × 10^−3^	5.92 × 10^−1^
F21	avg	−9.87	−3.47	−7.60	−4.54	−4.15	−9.8 × 10^−1^	−1.02 × 10^1^	−4.95
F22	min	−1.04 × 10^1^	−8.68	−1.04 × 10^1^	−6.98	−8.39	−2.87	−1.04 × 10^1^	−9.92
F22	std	4.39 × 10^−1^	1.56	3.08	6.69 × 10^−1^	1.77	4.24 × 10^−1^	7.72 × 10^−3^	1.29
F22	avg	−1 × 10^1^	−3.61	−7.40	−4.23	−4	−1.32	−1.04 × 10^1^	−4.97
F23	min	−1.05 × 10^1^	−6.99	−1.05 × 10^1^	−5.26	−9.29	−3.45	−1.05 × 10^1^	−1.05 × 10^1^
F23	std	1.38	1.18	3.39	5.87 × 10^−1^	2.50	6.19 × 10^−1^	6.29 × 10^−3^	1.73
F23	avg	−1 × 10^1^	−2.99	−6.32	−3.99	−4.16	−1.55	−1.05 × 10^1^	−5.40

**Table 3:** Test results of the moth algorithm improved by different strategies (D = 50).

		MIFSA	FSA	WOA	BOA	SWO	KOA	BWO	SABO
F1	min	0	0	1.03 × 10^−84^	1.10 × 10^−11^	1.37 × 10^−2^	4.72 × 10^4^	1.5 × 10^−270^	1.13 × 10^−200^
F1	std	0	1.53	8.99 × 10^−77^	8.51 × 10^−13^	5.94 × 10^3^	4.28 × 10^3^	0	0
F1	avg	0	2.86 × 10^−1^	4.49 × 10^−77^	1.31 × 10^−11^	1.23 × 10^3^	5.66 × 10^4^	8.91 × 10^−258^	2.28 × 10^−196^
F2	min	0	0	1.12 × 10^−59^	1.87 × 10^−9^	1.5 × 10^−2^	1.39 × 10^7^	7.36 × 10^−138^	3.14 × 10^−113^
F2	std	0	3.24 × 10^−1^	5.38 × 10^−49^	1.36 × 10^−9^	8.78	4.24 × 10^10^	1.97 × 10^−131^	6.79 × 10^−111^
F2	avg	0	8.60 × 10^−2^	9.9 × 10^−50^	4.50 × 10^−9^	3.67	1.98 × 10^10^	6.71 × 10^−132^	4.07 × 10^−111^
F3	min	0	0	2.75 × 10^4^	1.17 × 10^−11^	1.08	4.79 × 10^4^	1.49 × 10^−255^	4.3 × 10^−81^
F3	std	0	9.13 × 10^1^	9.36 × 10^3^	8.03 × 10^−13^	1.26 × 10^4^	2.01 × 10^4^	0	3.55 × 10^−43^
F3	avg	0	1.88 × 10^1^	4.24 × 10^4^	1.31 × 10^−11^	5.51 × 10^3^	8.62 × 10^4^	2.29 × 10^−243^	6.51 × 10^−44^
F4	min	0	0	2.63	5.27 × 10^−9^	7.39 × 10^−2^	7.35 × 10^1^	1.03 × 10^−132^	7.96 × 10^−79^
F4	std	0	4.23 × 10^−1^	2.54 × 10^1^	4.02 × 10^−10^	7.37	3.47	1.31 × 10^−126^	5.6 × 10^−77^
F4	avg	0	8.32 × 10^−2^	5.08 × 10^1^	6.16 × 10^−9^	5.66	8.19 × 10^1^	3.77 × 10^−127^	3.36 × 10^−77^
F5	min	2.73 × 10^−4^	2.88 × 10^1^	2.75 × 10^1^	2.89 × 10^1^	2.9 × 10^1^	1.17 × 10^8^	3.70 × 10−9	2.73 × 10^1^
F5	std	3.19	7.97 × 10^−2^	3.3 × 10^−1^	2.42 × 10^−2^	3.23 × 10^5^	3.27 × 10^7^	1.77 × 10−4	3.98 × 10^−1^
F5	avg	1.98	2.9 × 10^1^	2.8 × 10^1^	2.89 × 10^1^	6.42 × 10^4^	1.83 × 10^8^	4.03 × 10^−5^	2.83 × 10^1^
F6	min	8.27 × 10^−3^	3.94	9.31 × 10^−2^	4.45	6.64	4.62 × 10^4^	1.03 × 10^−16^	1.44
F6	std	3.31 × 10^−1^	1.01	2.59 × 10^−1^	6.56 × 10^−1^	9.39 × 10^2^	4.64 × 10^3^	4.06 × 10^−14^	6 × 10^−1^
F6	avg	3.57 × 10^−1^	7.39	3.84 × 10^−1^	5.91	3.22 × 10^2^	5.69 × 10^4^	2.13 × 10^−14^	2.64
F7	min	3.12 × 10^−5^	8.52 × 10^−3^	1.84 × 10^−4^	8.33 × 10^−4^	1.35 × 10^−2^	4.72 × 10^1^	6.15 × 10^−6^	4.25 × 10^−6^
F7	std	3.06 × 10^−3^	9.51 × 10^−2^	4.98 × 10^−3^	4.87 × 10^−4^	4.17 × 10^−1^	1.38 × 10^1^	1.22 × 10^−4^	8.65 × 10^−5^
F7	avg	2.83 × 10^−3^	1.16 × 10^−1^	3.67 × 10^−3^	1.65 × 10^−3^	1.98 × 10^−1^	8.16 × 10^1^	1.38 × 10^−4^	1.17 × 10^−4^
F8	min	−1.26 × 10^4^	−9.71 × 10^3^	−1.26 × 10^4^	−3.18 × 10^3^	−5.27 × 10^3^	−5.42 × 10^3^	−1.26 × 10^4^	−3.65 × 10^3^
F8	std	7.54 × 10^2^	1.01 × 10^3^	1.73 × 10^3^	3.52 × 10^2^	5.02 × 10^2^	1.85 × 10^−12^	2.36 × 10^−9^	2.93 × 10^2^
F8	avg	−1.22 × 10^4^	−7.20 × 10^3^	−1.07 × 10^4^	−3.85 × 10^3^	−4.19 × 10^3^	−5.42 × 10^3^	−1.26 × 10^4^	−2.97 × 10^3^
F9	min	0	0	0	0	3.91 × 10^−4^	3.56 × 10^2^	0	0
F9	std	0	9.04 × 10^−1^	1.04 × 10^−14^	6.83 × 10^1^	6.17 × 10^1^	2.18 × 10^1^	0	0
F9	avg	0	2.43 × 10^−1^	1.89 × 10^−15^	2.63 × 10^1^	3.67 × 10^1^	4.03 × 10^2^	0	0
F10	min	4.44 × 10^−16^	4.44 × 10^−16^	4.44 × 10^−16^	4.85 × 10^−9^	1.12 × 10^−2^	2 × 10^1^	4.44 × 10^−16^	4 × 10^−15^
F10	std	0	6.23 × 10^−2^	2.76 × 10^−15^	4.89 × 10^−10^	2.56	7.23 × 10^−15^	0	0
F10	avg	4.44 × 10^−16^	2.13 × 10^−2^	4.47 × 10^−15^	5.94 × 10^−9^	3.1	2 × 10^1^	4.44 × 10^−16^	4 × 10^−15^
F11	min	0	0	0	1.66 × 10^−12^	2.73 × 10^−3^	4.10 × 10^2^	0	0
F11	std	0	6.60 × 10^−1^	3.49 × 10^−2^	1.78 × 10^−12^	1.73	4.27 × 10^1^	0	0
F11	avg	0	2.01 × 10^−1^	6.37 × 10^−3^	4.23 × 10^−12^	1.43	5.16 × 10^2^	0	0
F12	min	1.23 × 10^−7^	1.56 × 10^−1^	4.51 × 10^−3^	2.05 × 10^−1^	4.09 × 10^−1^	2.03 × 10^8^	1.86 × 10^−15^	6.37 × 10^−2^
F12	std	1.41 × 10^−2^	4.56 × 10^−1^	9.74 × 10^−1^	1.79 × 10^−1^	1.74 × 10^4^	8.28 × 10^7^	4.05 × 10^−14^	8.27 × 10^−2^
F12	avg	1.19 × 10^−2^	1.35	2.07 × 10^−1^	6.61 × 10^−1^	3.28 × 10^3^	3.59 × 10^8^	3.43 × 10^−14^	2.19 × 10^−1^
F13	min	6.82 × 10^−7^	9.59 × 10^−1^	1.55 × 10^−1^	2.05	3.00	4.04 × 10^8^	1.47 × 10^−15^	1.61
F13	std	1.19 × 10^−1^	1.49	2.69 × 10^−1^	2.52 × 10^−1^	3.81 × 10^4^	1.72 × 10^8^	3.06 × 10^−13^	3.22 × 10^−1^
F13	avg	1.18 × 10^−1^	2.50	5.25 × 10^−1^	2.83	6.97 × 10^3^	7.72 × 10^8^	1.85 × 10^−13^	2.89
F14	min	9.98 × 10^−1^	9.98 × 10^−1^	9.98 × 10^−1^	9.98 × 10^−1^	9.99 × 10^−1^	2.01	9.98 × 10^−1^	1.00
F14	std	2.19	3.44	3.83	6.27 × 10^−1^	4.12	4.54	2.54 × 10^−6^	3.04
F14	avg	2.7	5.49	3.84	1.41	5.26	9.00	9.98 × 10^−1^	3.79
F15	min	3.50 × 10^−4^	1.50 × 10^−3^	3.08 × 10^−4^	3.10 × 10^−4^	6.21 × 10^−4^	5.68 × 10^−3^	3.10 × 10^−4^	3.17 × 10^−4^
F15	std	4.01 × 10^−3^	1.04 × 10^−2^	5.23 × 10^−4^	6.61 × 10^−5^	7.92 × 10^−3^	1.50 × 10^−2^	5.17 × 10^−5^	1.18 × 10^−3^
F15	avg	3.57 × 10^−3^	1.14 × 10^−2^	7.83 × 10^−4^	3.96 × 10^−4^	7.14 × 10^−3^	3.08 × 10^−2^	3.64 × 10^−4^	8.93 × 10^−4^
F17	min	3.98 × 10^−1^	3.98 × 10^−1^	3.98 × 10^−1^	3.98 × 10^−1^	3.98 × 10^−1^	3.98 × 10^−1^	3.98 × 10^−1^	3.98 × 10^−1^
F17	std	3.62 × 10^−6^	2.28 × 10^−2^	1.11 × 10^−5^	5.46 × 10^−3^	3.29 × 10^−2^	1.29 × 10^−1^	1.51 × 10^−3^	1.91 × 10^−1^
F17	avg	3.98 × 10^−1^	4.20 × 10^−1^	3.98 × 10^−1^	4.01 × 10^−1^	4.28 × 10^−1^	5.49 × 10^−1^	4.00 × 10^−1^	4.91 × 10^−1^
F18	min	3.00	3.00	3.00	3.00	3.00	3.27	3.02	3.00
F18	std	1.30 × 10^−6^	3.14	1.29 × 10^−4^	1.84 × 10^−1^	2.08 × 10^−1^	1.11 × 10^1^	6.25 × 10^−1^	1.75
F18	avg	3.00	4.70	3.00	3.19	3.14	1.25 × 10^1^	3.74	3.80
F19	min	−3.86	−3.86	−3.86	−3.84	−3.86	−3.86	−3.86	−3.86
F19	std	2.93 × 10^−2^	7.57 × 10^−2^	7.54 × 10^−3^	2.04 × 10^−1^	1.37 × 10^−2^	9.64 × 10^−2^	3.03 × 10^−3^	1.96 × 10^−1^
F19	avg	−3.85	−3.79	−3.86	−3.96	−3.85 ×	−3.70	−3.86	−3.66
F20	min	−3.32	−3.06	−3.32	−2.17	−3.31	−2.90	−3.31	−3.32
F20	std	1.05 × 10^−1^	3.46 × 10^−1^	9.19 × 10^−2^	5.97	1.55 × 10^−1^	3.78 × 10^−1^	3.13 × 10^−2^	2.04 × 10^−1^
F20	avg	−3.19	−2.70	−3.24	−4.00	−3.09	−2.40	−3.29	−3.18
F21	min	−1.02 × 10^1^	−8.22	−1.02 × 10^1^	−6.56	−9.32	−3.27	−1.02 × 10^1^	−8.26
F21	std	2.09 × 10^−1^	1.57	2.58	5.05 × 10^−1^	2.20	6.19 × 10^−1^	8.43 × 10^−3^	8.54 × 10^−1^
F21	avg	−9.98	−3.82	−8.34	−4.47	−3.65	−1.14	−1.01 × 10^1^	−4.91
F22	min	−1.04 × 10^1^	−5.53	−1.04 × 10^1^	−5.03	−8.12	−3.32	−1.04 × 10^1^	−6.41
F22	std	2.30 × 10^−1^	1.25	3.00	5.09 × 10^−1^	1.47	6.68 × 10^−1^	2.35 × 10^−2^	5.06 × 10^−1^
F22	avg	−1.02 × 10^1^	−3.30	−7.47	−4.24	−3.30	−1.38	−1.04 × 10^1^	−4.83
F23	min	−1.05 × 10^1^	−8.68	−1.05 × 10^1^	−5.59	−7.43	−4.23	−1.05 × 10^1^	−9.40
F23	std	5.68 × 10^−1^	1.54	3.31	7.85 × 10^−1^	1.54	6.80 × 10^−1^	1.32 × 10^−2^	1.66
F23	avg	−1.02 × 10^1^	−3.44	−7.89	−4.20	−4.07	−1.60	−1.05 × 10^1^	−5.30

**Table 4:** Coverage Optimization Results of 8 Algorithms.

Iteration	MIFSA	FSA	SABO	WOA	BOA	SWO	KOA	BWO
50	85.64%	80.72%	79.56%	79.88%	79.4%	77.96%	79%	79.12%
100	88.12%	80.64%	79.32%	80.08%	81.36%	80.04%	79.64%	84.60%
150	87.36%	81%	77.16%	77.36%	82.4%	81.52%	77.4%	86.56%
200	88.64%	82.96%	79.64%	81%	80.76%	80.64%	81.92%	85.68%

## Data Availability

The data that support the findings of this study are available from the corresponding author upon request. There are no restrictions on data availability.
